# Magnesium depletion score is associated with arterial stiffness: data from the Brisighella Heart Study

**DOI:** 10.1097/HJH.0000000000004253

**Published:** 2026-02-10

**Authors:** Federica Fogacci, Marina Giovannini, Elisa Grandi, Sergio D’Addato, Claudio Borghi, Arrigo F.G. Cicero

**Affiliations:** a Hypertension and Cardiovascular Risk Research Center, Medical and Surgical Sciences Department, Alma Mater Studiorum University of Bologna; b Cardiovascular Medicine Unit, Heart, Chest and Vascular Department, IRCCS Azienda Ospedaliero-Universitaria di Bologna, Bologna, Italy

**Keywords:** arterial stiffness, augmentation index, epidemiology, magnesium, pulse wave velocity

## Abstract

**Background and aims:**

The magnesium depletion score (MDS) estimates magnesium deficiency risk by integrating dietary intake and physiological losses. This study evaluated the association between MDS and arterial stiffness in a rural Mediterranean population.

**Methods:**

We analyzed data from 2048 participants (49.2% men, 50.8% women) in the Brisighella Heart Study. MDS and arterial stiffness parameters – augmentation index (AIx) and carotid–femoral pulse wave velocity (cfPWV) – were assessed using validated methods. Multiple regression models adjusted for age and mean arterial pressure included sex, smoking, physical activity, BMI, heart rate, fasting glucose, low-density lipoprotein cholesterol (LDL-C), triglycerides, serum uric acid, estimated glomerular filtration rate (eGFR), and MDS.

**Results:**

An MDS at least 2 was observed in 51.6% of participants, more often in men (*P* < 0.001). Higher MDS was significantly associated with increased AIx and cfPWV in both sexes (*P* < 0.001). MDS remained an independent predictor of AIx (*β* = 0.087, *P* = 0.011) and cfPWV (*β* = 0.131, *P* = 0.013) after adjustment.

**Conclusion:**

Higher MDS values correlate with greater arterial stiffness, suggesting that magnesium imbalance may negatively affect vascular health.

## INTRODUCTION

Magnesium is a metal with high reducing power, predominantly found in nuts, whole grains, cocoa, certain spices, and vegetables [1]. It plays a critical role in numerous metabolic pathways, serving as an essential cofactor in over 600 enzymatic reactions. In addition, magnesium is a key regulator of cellular ion channels, membrane transporters, and signaling mechanisms that govern the transmembrane movement of calcium, potassium, and sodium. Consequently, it serves as a fundamental modulator of cardiac excitability, smooth muscle contractility, vasomotor tone, and ultimately, systemic blood pressure regulation [2].

Magnesium deficiency has been associated with elevated levels of pro-inflammatory cytokines – including tumor necrosis factor-alpha (TNF-α), interleukin-6 (IL-6), and interleukin-1β (IL-1β) – as well as acute-phase proteins such as fibrinogen, α2- macroglobulin, and complement. Furthermore, magnesium deficiency has been shown to activate phagocytic immune cells, increase intracellular calcium influx, stimulate *N*-methyl-D-aspartate (NMDA) receptors and the NF-κB signaling pathway, promote gut dysbiosis, and accelerate cellular senescence, collectively contributing to a state of chronic inflammatory stress [3].

A recent meta-analysis of 19 cohort studies, encompassing 1 168 756 individuals and follow-up durations ranging from 3.5 to 32 years, reported 52 378 all-cause deaths, 23 478 cardiovascular deaths, and 11 408 cancer-related deaths. Higher dietary magnesium intake was significantly associated with a reduced risk of all-cause mortality [pooled effect size (ES): 0.87; 95% confidence interval (CI): 0.79–0.97; *P* = 0.009; *I*^2^ = 70.7%; *P* for heterogeneity <0.001] and cancer-related mortality (pooled ES: 0.80; 95% CI: 0.67–0.97; *P* = 0.023; *I*^2^ = 55.7%; *P* = 0.027). However, no significant association was observed between dietary magnesium intake and cardiovascular mortality (pooled ES: 0.93; 95% CI: 0.82– 1.07; *P* = 0.313; *I*^2^ = 72.3%; *P* < 0.001) [4].

Notably, serum magnesium concentrations appear to be more robustly and consistently associated with cardiovascular outcomes compared to dietary intake estimates. A meta-analysis of 18 prospective cohort studies, including 544 581 participants and 22 658 cardiovascular events, demonstrated that individuals with higher (within normal range) serum magnesium levels had a significantly lower risk of cardiovascular disease [relative risk (RR): 0.64; 95% CI: 0.51–0.80] and coronary heart disease (RR: 0.70; 95% CI: 0.57– 0.85), compared to those with the lowest levels. Moreover, each 0.1 mg/dl increase in serum magnesium was associated with a reduced risk of cardiovascular disease (RR: 0.93; 95% CI: 0.88–0.97) and coronary heart disease (RR: 0.90; 95% CI: 0.84–0.96). Dose–response analyses confirmed linear inverse associations with no evidence of nonlinearity for cardiovascular (*P* = 0.833) or coronary outcomes (*P* = 0.193) [5].

The discrepancy between dietary magnesium intake and serum magnesium levels in relation to cardiovascular risk may be attributed to substantial variability in magnesium consumption over time and the challenges of accurately assessing intake via dietary recall or food frequency questionnaires. In contrast, serum magnesium concentrations may provide a more comprehensive reflection of both intake and physiological losses.

To address the limitations of dietary estimation, the magnesium depletion score (MDS) has been developed as a practical and validated tool to estimate magnesium status, incorporating both dietary and nondietary factors contributing to magnesium depletion [6]. Unlike serum magnesium concentrations – which can appear normal even in the presence of significant intracellular depletion or chronic loss – MDS offers a broader, integrative assessment of magnesium status over time. This may be particularly relevant in routine clinical practice, where serum magnesium measurements are not always performed and can be influenced by transient or acute-phase factors. The MDS incorporates chronic contributors to magnesium loss, such as impaired renal function, medication use, and alcohol consumption, thereby reflecting both intake and ongoing physiological depletion. Moreover, it may serve as a more accessible tool in large-scale epidemiological assessments or primary care settings where biochemical testing is not readily available.

In this context, the present study aimed to investigate the association between MDS and arterial stiffness – an established marker of vascular aging and cardiovascular risk – in a rural Mediterranean population. By doing so, we sought to determine whether the MDS could offer clinically relevant insights into cardiovascular health beyond what is provided by serum magnesium concentrations alone.

## METHODS

The methodology of the Brisighella Heart Study has been extensively described in previous publications [7,8]. In summary, it is the longest running epidemiological study in Europe, initiated in 1972, and based on a randomized sample representative of the rural population of Brisighella, a small town in Northern Italy. At baseline, the study enrolled 2939 adult Caucasian participants (1491 men and 1448 women), all free from cardiovascular disease. Participants have undergone clinical evaluations every 4 years since the study's inception, with data collected on clinical parameters, biochemical markers, morbidity, and mortality.

The study complies with the principles outlined in the Declaration of Helsinki and its subsequent amendments. The protocol was approved by the Ethical Committee of the IRCCS Azienda Ospedaliero-Universitaria di Bologna (Code: BrixFollow-up_1972–2024), and all participants provided written informed consent.

At each follow-up visit, participants undergo comprehensive assessments including detailed medical history, physical examination, and evaluation of lifestyle factors such as dietary habits, smoking status, and current medications. Anthropometric measurements, resting blood pressure (BP), heart rate, and a 12-lead electrocardiogram are also recorded [9].

Dietary intake is assessed using a validated semi-quantitative tool – the Dietary Quality Index – which collects information on food consumption over the preceding 12 months [10]. Anthropometric data (height, weight, and waist circumference) are obtained using standardized procedures. BMI is calculated as weight (kg) divided by height squared (m^2^) and treated as a continuous variable.

Blood pressure (systolic and diastolic) is measured three times at 1-min intervals after the participant has been seated and resting for at least 5 min. The average of the three readings is used, in accordance with international guidelines [11]. Pulse pressure (PP) is calculated as the difference between SBP and DBP, while mean arterial pressure (MAP) is calculated as DBP + 1/3 (SBP − DBP). Hypertension is defined as SBP at least 140 mmHg, DBP at least 90 mmHg, or current use of antihypertensive medication.

Noninvasive assessments of central BP, augmentation index (AIx – a marker of arterial stiffness derived from aortic pressure waveforms), and carotid–femoral pulse wave velocity (cfPWV) are performed using the Vicorder system (Skidmore Medical Ltd., Bristol, UK), an oscillometric device validated for epidemiological studies and known for high inter-operator and intra-operator reliability [12,13]. Central BP parameters are derived from brachial waveforms, calibrated using brachial SBP and DBP, and processed via a validated brachial-to-aortic transfer function [14,15].

Fasting blood samples are collected to assess routine laboratory parameters, including total cholesterol (TC), triglycerides, high-density lipoprotein cholesterol (HDL-C), apolipoprotein B100 and AI, lipoprotein (a) [Lp(a)], fasting plasma glucose (FPG), gamma- glutamyl transferase (gGT), creatinine, and serum uric acid (SUA). Low-density lipoprotein cholesterol (LDL-C) is calculated using the Friedewald formula for triglycerides less than 400 mg/dl. Estimated glomerular filtration rate (eGFR) is calculated using the CKD-EPI formula. All laboratory analyses are performed using standardized protocols by trained personnel [7].

For the present analysis, we included data from 2048 participants [1007 men (49.2%) and 1041 women (50.8%)] for whom both the MDS and arterial stiffness parameters were available, and after exclusion of those subjects assuming mineralcorticoid receptor agonists (*N* = 7) and those ones currently taking magnesium dietary supplements (*N* = 11).

The MDS was calculated based on four components: current use of thiazides or loop diuretics (1 point), current use of proton pump inhibitors (1 point), reduced kidney function (eGFR <90 ml/min = 1 point; eGFR <60 ml/min = 2 points), and heavy alcohol consumption (1 point). Heavy alcohol consumption was defined according to standard epidemiological criteria as more than one alcoholic drink per day for women or more than two drinks per day for men [16].

Continuous variables are expressed as means ± standard deviation (SD), and categorical variables as absolute frequencies and percentages. The Kolmogorov–Smirnov test was used to assess the normality of continuous variables. Comparisons by sex or coffee intake were conducted using the chi-square test for categorical variables and analysis of variance (ANOVA) for continuous variables, followed by Tukey's post hoc test. Nonnormally distributed variables were log-transformed before analysis. Spearman's rank correlation was used to assess the relationship between the MDS and other continuous variables. Multiple regression analyses were then performed to identify the strongest predictors of AIx and cfPWV, in models adjusted for age and MAP. Predictor variables included sex, smoking status, physical activity, BMI, heart rate, FPG, LDL-C, triglycerides, SUA, eGFR, and MDS. Given the distinct distribution patterns of arterial stiffness parameters and predictors, stratified analyses by sex were also conducted. All statistical analyses were performed using SPSS version 28.0 for Windows (IBM Corp., Armonk, New York, USA), with *P* values less than 0.05 considered statistically significant.

## RESULTS

The main characteristics of the study population are summarized in Table 1.

**TABLE 1:** Main characteristics of the studied population sample by sex

Parameters	Men (*N *=* *1007)	Women (*N *=* *1048)	*P* value
Age (years)	59.3 ± 15.4	59.8 ± 15.3	0.425
Waist circumference (cm)	96.9 ± 11.6	88.1 ± 13.5	<0.001
BMI (kg/m2)	27.1 ± 3.9	26.3 ± 5.3	<0.001
SBP (mmHg)	141.3 ± 9.9	141.5 ± 11.2	0.826
DBP (mmHg)	75.4 ± 5.7	71.5 ± 5.5	<0.001
Pulse pressure (mmHg)	65.9 ± 7.8	69.9 ± 7.4	<0.001
Augmentation Index	24.7 ± 8.5	27.1 ± 9.4	<0.001
Pulse wave velocity (m/s)	9.4 ± 2.8	9.2 ± 2.8	0.129
Heart rate (bpm)	61.8 ± 11.3	66.4 ± 11.3	<0.001
Total cholesterol (mg/dl)	211.4 ± 29.5	221.7 ± 29.6	<0.001
LDL cholesterol (mg/dl)	137.3 ± 25.8	142.9 ± 26.9	<0.001
HDL cholesterol (mg/dl)	49.6 ± 13.8	56.4 ± 15.3	<0.001
Triglycerides (mg/dl)	125.9 ± 43.2	113.1 ± 29.7	<0.001
Lipoprotein (a) (mg/dl)	19.9 ± 26.8	23.3 ± 29.7	<0.001
Apolipoprotein B (mg/dl)	92.2 ± 19.9	93.9 ± 22.6	<0.001
Apolipoprotein A (mg/dl)	148.4 ± 25.0	163.9 ± 29.9	<0.001
Fasting plasma glucose (mg/dl)	99.5 ± 10.7	94.1 ± 7.1	<0.001
Serum uric acid (mg/dl)	5.8 ± 1.3	4.6 ± 1.1	<0.001
gamma-Glutamyl-transferase (mg/dl)	34.4 ± 15.5	21.8 ± 11.2	<0.001
estimated GFR (ml/min)	72.8 ± 15.2	69.3 ± 15.9	<0.001

No significant differences were observed between men and women in terms of age, SBP, or cfPWV. An eGFR below 60 ml/min was found in 191 men (19.0%) and 286 women (27.5%), while an eGFR between 60 and 90 ml/min was observed in 686 men (68.1%) and 656 women (63%).

Heavy alcohol consumption was reported by 56 men (6.8%) and 26 women (3.2%). Diuretic use was documented in 50 men (6.6%) and 72 women (9.1%), whereas proton pump inhibitors were used by 99 men and 101 women. An MDS at least 2 was recorded in 51.6% of the total study population.

The sex-specific distribution of MDS is shown in Table 2. Lower MDS values were more frequently observed in women than in men (Pearson's chi-square = 44.313, degrees of freedom = 3, *P* < 0.001).

**TABLE 2:** Distribution of magnesium depletion score between sexes in the considered population sample

MDS	Men	Women	Total
0	75 (7.4%)	78 (7.5%)	153 (7.5%)
1	341 (33.9%)	498 (47.8%)	839 (40.9%)
2	446 (44.3%)	343 (32.9%)	789 (38.5%)
>2	145 (14.4%)	122 (11.7%)	267 (13.1%)
Total	1007 (100%)	1041 (100%)	2048 (100%)

GFR, glomerular filtration rate; HDL, high-density lipoprotein; LDL, low-density lipoprotein; *N*, number of individuals.

The main characteristics of the study population stratified by MDS are presented in Table 3.

**TABLE 3:** Distribution of available continuous parameters in the study population according to magnesium depletion score categories

Parameter	Magnesium Depletion Score				
	Score 0	Score 1	Score 2	Score >2	*P*
Age (years)	43.5 ± 13.0	53.9 ± 14.1	63.9 ± 13.2	73.4 ± 9.2	<0.001
Waist circumference (cm)	59.6 ± 15.3	88.5 ± 17.7	95.1 ± 12.8	96.9 ± 11.9	<0.001
BMI (kg/m2)	25.9 ± 5.3	26.1 ± 4.9	27.1 ± 4.3	27.8 ± 4.1	<0.001
SBP (mmHg)	132.9 ± 9.8	138.1 ± 9.9	143.9 ± 10.4	149.1 ± 10.9	<0.001
DBP (mmHg)	71.2 ± 5.3	72.6 ± 5.7	74.3 ± 5.8	74.6 ± 5.6	<0.001
PP (mmHg)	61.7 ± 7.9	65.5 ± 7.1	69.6 ± 8.6	74.4 ± 9.0	<0.001
Augmentation Index	21.6 ± 8.8	25.1 ± 9.8	26.9 ± 7.6	27.8 ± 9.8	<0.001
Pulse wave velocity (m/s)	8.1 ± 1.7	9.0 ± 2.6	9.5 ± 2.8	10.4 ± 3.4	<0.001
Heart rate (bpm)	64.6 ± 12.3	65.1 ± 11.6	63.3 ± 11.0	63.5 ± 12.0	0.113
Total cholesterol (mg/dl)	206.8 ± 29.4	216.1 ± 28.0	219.4 ± 29.1	215.8 ± 28.8	0.084
LDL cholesterol (mg/dl)	136.2 ± 26.0	139.7 ± 24.1	142.1 ± 25.3	138.1 ± 26	0.168
HDL cholesterol (mg/dl)	50.96 ± 13.7	53.8 ± 15.1	52.8 ± 14.8	52.6 ± 15.9	0.134
Triglycerides (mg/dl)	103.6 ± 46.1	114.9 ± 32.5	125.4 ± 49.2	124.5 ± 35.4	0.041
Lipoprotein (a) (mg/dl)	17.3 ± 25.9	20.9 ± 28.5	23.2 ± 29.0	22.1 ± 27.1	0.113
Apolipoprotein B (mg/dl)	85.5 ± 20.4	90.9 ± 21.4	96.2 ± 20.7	94.6 ± 21.4	<0.001
Apolipoprotein A (mg/dl)	148.6 ± 23.9	156.3 ± 29.6	157.5 ± 28.1	156.9 ± 29.4	0.007
Fasting plasma glucose (mg/dl)	93.3 ± 13.5	93.5 ± 8.8	99.3 ± 10.3	101.5 ± 11.7	<0.001
Serum uric acid (mg/dl)	4.7 ± 1.2	4.9 ± 1.2	5.4 ± 1.5	5.7 ± 1.3	<0.001
Estimated GFR (ml/min)	98.9 ± 8.5	76.1 ± 10.7	66.4 ± 13.1	53.5 ± 9.4	<0.001

BP, blood pressure; GFR, glomerular filtration rate; HDL, high-density lipoprotein; LDL, low-density lipoprotein.

**FIGURE 1:**
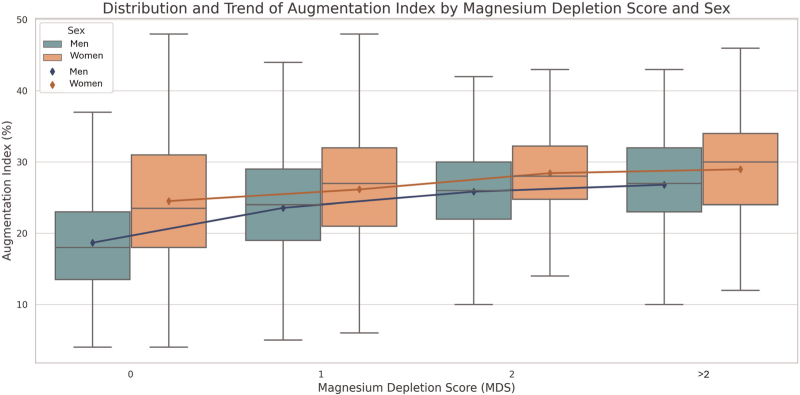
Distribution of augmentation index by sex and magnesium depletion score.

**FIGURE 2:**
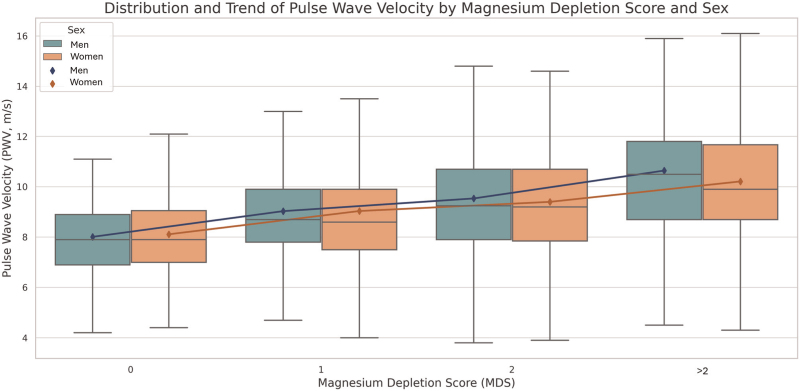
Distribution of carotid–femoral pulse wave velocity by sex and magnesium depletion score.

MSD increases with age, waist circumference, BMI, blood pressure, serum triglycerides, and uric acid, while it decreases with eGFR. Both AIx and cfPWV increased progressively with higher MDS values. The distribution of AIx and cfPWV by sex and MDS category is illustrated in Figs. 1 and 2. In both men and women, a significant upward trend in these arterial stiffness parameters was observed with increasing MDS (*P* < 0.001).

The prevalence of antihypertensive, lipid-lowering and glucose-lowering therapy increased with higher MDS values, in parallel with the less favorable cardiometabolic profile of these groups. The prevalence of antihypertensive, lipid-lowering and glucose-lowering therapies increased with higher MDS values, in line with the less favorable cardiometabolic profile observed in these groups. Nevertheless, meaningful comparisons of MDS categories based on drug distribution are not possible, as these agents are used in varying combinations, dosages and treatment durations, and achieve differing degrees of risk-factor control.

In an age-adjusted and MAP-adjusted multiple regression model – including sex, smoking habits, physical activity, BMI, heart rate, FPG, LDL-C, TG, SUA and MDS as predictors – the main determinants of AIx in the overall population were apolipoprotein B (Apo B; *B* = 0.311, 95% CI: 0.099–0.551, *β* = 0.211, *P* < 0.001), SUA (*B* = 0.117, 95% CI: 0.054–0.237, *β* = 0.108, *P* < 0.001), and MDS (*B* = 0.099, 95% CI: 0.023–0.144, *β* = 0.087, *P* = 0.011).

Similarly, the main predictors of cfPWV were Apo B (*B* = 0.172, 95% CI: 0.058–0.272, *β* = 0.367, *P* < 0.001), SUA (*B* = 0.134, 95% CI: 0.019–0.231, *β* = 0.148, *P* < 0.001), and MDS (*B* = 0.116, 95% CI: 0.018–0.131, *β* = 0.131, *P* = 0.013). When analyses were stratified by sex, the same predictors for both AIx and cfPWV were confirmed in men and women.

To address the possibility that the association between MDS and arterial stiffness might simply reflect the relationship between arterial stiffness and adiposity, BMI was included as a covariate in all multivariable regression models evaluating AIx and cfPWV. In these models, MDS remained an independent predictor of both arterial stiffness parameters after adjustment for BMI, age, mean arterial pressure and other potential confounders.

In additional sensitivity analyses in which waist circumference was entered instead of BMI, the association between MDS and arterial stiffness was materially unchanged (data not shown).

## DISCUSSION

In our cohort, instrumental markers of arterial stiffness – namely AIx and cfPWV – were directly proportional to MDS values in both sexes. The MDS is a simple and practical index originally developed and validated in a large cohort of the National Health and Nutrition Examination Survey (NHANES) by Fan *et al.* in 2021 [16]. In the NHANES cohort, MDS was associated with an increased risk of all-cause and cardiovascular disease mortality in a dose–response manner, particularly when low magnesium intake occurred in conjunction with a high MDS [16].

Moreover, MDS has been linked to various chronic conditions – including osteoporosis [17], kidney stones [18], gout [19], metabolic dysfunction-associated steatotic liver disease (MASLD) [20], type 2 diabetes and diabetic retinopathy [21,22], depression [23], chronic obstructive pulmonary disease [24], periodontitis [25], sleep apnea [26], and heart failure [27] – most of which are closely associated with increased cardiovascular risk.

Our findings support and extend this evidence by demonstrating a strong association between higher MDS values and markers of arterial aging. Specifically, both AIx and cfPWV increased significantly across MDS categories in both sexes, and multiple regression analysis confirmed MDS as an independent predictor of both parameters – two widely accepted noninvasive measures of vascular aging [28].

These results are particularly relevant in light of recent NHANES data linking MDS to abdominal aortic calcification (AAC) and hypertension. In the 2007–2018 NHANES cohort (*N* = 9708), multivariable logistic regression revealed that each unit increase in MDS was associated with an 87% greater risk of hypertension [odds ratio (OR) = 1.87, 95% CI: 1.64–2.13], after adjustment for confounding factors. Participants in the highest MDS category had an over eightfold increased risk of hypertension compared to those in the lowest (OR = 8.3, 95% CI: 4.8–14.4; *P* < 0.001) [29]. Furthermore, in a separate analysis of 12 485 NHANES participants (including 2537 all-cause deaths and 707 cardiovascular deaths), individuals with higher MDS had the highest rates of both all-cause and cardiovascular mortality (*P* < 0.001), with the highest MDS group showing a 60% increased risk of cardiovascular death (hazard ratio = 1.6, 95% CI: 1.2–2.2) compared to those with the lowest MDS [30].

In another subset of 2640 NHANES participants with available AAC data (mean AAC score: 1.5 ± 0.1), higher MDS levels were associated with a clear trend toward increased AAC scores. Specifically, AAC scores rose progressively from MDS 0 to MDS at least 4, reaching 4.99 (95% CI: 3.49–6.49) in the highest category. Compared with MDS 0, those with MDS at least 4 had significantly higher AAC scores (*β* = 4.2, 95% CI: 2.8–5.7; *P* < 0.001) [31].

It is plausible that MDS increases with age, as older individuals are more likely to be prescribed medications such as diuretics and proton pump inhibitors and may also present with decreased kidney function. Given that arterial stiffness also tends to rise with age, this could represent a confounding factor. However, our regression models were adjusted for age to address this issue.

Participants with higher MDS values were not only older but also exhibited higher BMI and waist circumference, consistent with a clustering of magnesium-depleting conditions in individuals with an adverse cardiometabolic profile. To minimize confounding by adiposity, all regression models were adjusted for BMI, and sensitivity analyses additionally accounting for waist circumference yielded similar results, indicating that the association between MDS and arterial stiffness is not merely explained by general or central obesity.

Overall, our findings reinforce the importance of promoting adequate magnesium intake through a balanced diet and, when appropriate, targeted supplementation [32]. However, other contributors to MDS must also be addressed. Reducing alcohol consumption should be actively encouraged [33], and the rational prescribing of proton pump inhibitors should be improved to avoid unnecessary long-term use [34].

While thiazide and thiazide-like diuretics are well established antihypertensive agents – particularly effective in lowering systolic and pulse pressure in older individuals – their use may contribute to magnesium depletion, especially when prescribed long-term. In patients with additional magnesium-wasting conditions or elevated MDS, alternative first-line therapies, such as RAAS inhibitors or calcium channel blockers, may be considered to minimize further electrolyte imbalance. Treatment decisions should be individualized based on the overall cardiovascular profile, electrolyte status and comorbidities [35]. More broadly, optimizing all modifiable risk factors for chronic kidney disease remains a public health priority [36].

Despite the strength of our findings, one limitation of the present study is the lack of available serum magnesium concentrations, which prevented a direct comparison between the MDS and biochemical magnesium status. Nevertheless, this absence does not undermine the utility of the MDS. Serum magnesium reflects less than 1% of total body magnesium and is homeostatically regulated, often remaining within normal limits even in individuals with clinically relevant magnesium deficiency. Therefore, serum levels may underestimate chronic or intracellular magnesium depletion [37]. In addition, we did not have data on serum potassium, and we, therefore, could not assess whether hypokalemia **–** which frequently accompanies magnesium deficiency and reflects increased renal potassium wasting – might act as an intermediate phenotype linking MDS to vascular damage [38,39]. Low potassium levels have been associated with increased arterial stiffness in specific high-risk conditions, such as primary aldosteronism, where serum potassium is an independent correlate of augmentation index, suggesting that our findings should be interpreted as capturing the overall burden of magnesium-related electrolyte disturbances, rather than the isolated effect of magnesium alone [40]. In contrast, the MDS incorporates multiple chronic depletion mechanisms – such as reduced kidney function, diuretic and proton pump inhibitor use, and excessive alcohol consumption – providing a broader and potentially more clinically meaningful estimate of magnesium-related physiological burden. This integrative nature may explain the consistent association of MDS with arterial stiffness in our cohort.

We also acknowledge that the medication use in the Brisighella Heart Study was self- reported, which may introduce reporting bias. However, interviews were conducted by trained personnel to enhance data accuracy. Furthermore, the specific characteristics of our cohort – namely its rural setting, long-standing dietary patterns, and relative genetic homogeneity – may limit the generalizability of these findings to other Italian or European populations.

Despite these limitations, this is the first study to report a positive association between MDS and arterial stiffness in an Italian rural population. Previous studies have predominantly focused on North American populations.

In conclusion, MDS is significantly associated with increased arterial stiffness in a well characterized rural Mediterranean population, supporting its potential role as a noninvasive marker of vascular aging.

## ACKNOWLEDGEMENTS

The authors express their sincere gratitude to the general practitioners of Brisighella, the citizens and the Mayor of Brisighella, as well as the Faenza Public Health District, for their invaluable collaboration and ongoing support.

The Brisighella Heart Study Group: Arrigo F.G. Cicero, Sergio D’Addato, Elisa Grandi, Federica Fogacci, Elisabetta Rizzoli, Marina Giovannini, Fulvio Ventura, Pierangelo Coppola, Ilaria Ricci Iamino, Matteo Landolfo, Silvia Palmisano, Martina Rosticci, Giuseppe Derosa, Stefano Bacchelli, Claudio Borghi.

Author contributions: conceptualization: A.F.G.C.; data curation: F.F., M.G., E.G., S.D.A., C.B. and A.F.G.C.; formal analysis: A.F.G.C.; funding acquisition: C.B. and A.F.G.C.; investigation: F.F., M.G., E.G., S.D.A. and A.F.G.C.; methodology: F.F. and A.F.G.C.; project administration: C.B. and A.F.G.C.; supervision: C.B. and A.F.G.C.; visualization: F.F. and M.G.; roles/writing – original draft: F.F. and A.F.G.C.; writing – review and editing: M.G., E.G., S.D.A. and C.B.

Funding: this research was supported by institutional funding from the University of Bologna (RFO 2015 and RFO 2016), and by the National Recovery and Resilience Plan (NRRP), Mission 4, Component 2, Investment 1.3—Call for Proposals No. 341 dated 15 March 2022, issued by the Italian Ministry of University and Research and funded by the European Union – NextGenerationEU. The study was conducted within the framework of the project ‘ON Foods – Research and Innovation Network on Food and Nutrition Sustainability, Safety and Security – Working ON Foods’ (Project Code: PE00000003; Concession Decree No. 1550 dated 11 October 2022, Italian Ministry of University and Research).

### Conflicts of interest

There are no conflicts of interest.
